# A Comparison of Medical Birth Register Outcomes between Maternity Health Clinics and Integrated Maternity and Child Health Clinics in Southwest Finland

**DOI:** 10.5334/ijic.2024

**Published:** 2016-07-08

**Authors:** Miia Tuominen, Anne Kaljonen, Pia Ahonen, Juha Mäkinen, Päivi Rautava

**Affiliations:** PhD Student, Department of Clinical Medicine, Public Health, University of Turku and Institute for Child and Youth Research, University of Turku, FI-20014 Turun yliopisto, Turku, Finland; Statistician, Institute for Child and Youth Research, University of Turku, FI-20014 Turun yliopisto, Turku, Finland; Head of Education and Research, Faculty of Health and Well-being, Turku University of Applied Sciences, Ruiskatu 8, 20760 Turku, Finland; Professor, Chief physician, Department of Obstetrics and Gynaecology, University of Turku and Department of Obstetrics and Gynaecology, Turku University Hospital, PO Box 52, 20521 Turku, Finland; Professor, Chief physician of research, Public Health Department, University of Turku and Turku Clinical Research Centre, Turku University Hospital, PO Box 52, 20521 Turku, Finland

**Keywords:** maternal health services, prenatal care, STEPS study, comparative study, maternal outcomes, perinatal outcomes

## Abstract

**Introduction:**

Primary maternity care services are globally provided according to various organisational models. Two models are common in Finland: a maternity health clinic and an integrated maternity and child health clinic. The aim of this study was to clarify whether there is a relation between the organisational model of the maternity health clinics and the utilisation of maternity care services, and certain maternal and perinatal health outcomes.

**Methods:**

A comparative, register-based cross-sectional design was used. The data of women (N = 2741) who had given birth in the Turku University Hospital area between 1 January 2009 and 31 December 2009 were collected from the Finnish Medical Birth Register. Comparisons were made between the women who were clients of the maternity health clinics and integrated maternity and child health clinics.

**Results:**

There were no clinically significant differences between the clients of maternity health clinics and integrated maternity and child health clinics regarding the utilisation of maternity care services or the explored health outcomes.

**Conclusions:**

The organisational model of the maternity health clinic does not impact the utilisation of maternity care services or maternal and perinatal health outcomes. Primary maternity care could be provided effectively when integrated with child health services.

## Introduction

Effective maternity care services are globally produced according to varying organisational models, which are based on several evidence-based interventions and the national guidelines [1,2,3,4,5]. However, society-sensitive research is still needed to obtain evidence for implementing a standard organisational model for consistent and high-quality maternity care in each country [6]. Commonly used indicators for evaluating the maternity care produced in varying organisational settings are the health outcomes of the mother and baby and the utilisation of the services [7].

Primary maternity care in Finland is provided by the municipal maternity and child health clinic system that was mandated by law in 1944 to guarantee free health-care services for every pregnant woman and all children under the school age (age of seven). Today, maternity health clinics are part of communal health centres, responding to the need for health promotion and support for child-bearing families, and providing a way to monitor the pregnancy and early postpartum periods. Free screenings for foetal chromosome and growth defects during pregnancy are also offered to families. In the maternity health clinics there is also a particular emphasis on supporting parenthood and the welfare of the whole family [8,9,10]. Over 99.5% of child-bearing families are estimated to be users of these clinics [11].

The midwife-led models are effective and safe ways to produce maternity care services [12]. The Finnish maternity health clinics are led by public health nurses in close cooperation with general practitioners. Midwives are also able to work in maternity health clinics; however, they usually also have a public health nurse degree [13]. Tertiary level antenatal care and consultation for the maternity health clinics’ personnel are provided by hospital-based outpatient maternity clinics.

Finland has, in common with the other Nordic countries, high levels of maternal and perinatal health. For example, the perinatal and maternal mortality rates are among of the lowest in the world [11,14,15]. In a European comparison, Finnish rates for low birth weight and preterm births are relatively low and the caesarean section rate is among the lowest [15]. The main risk factors for perinatal health in Finland are smoking during pregnancy and mothers being overweight before and during pregnancy [11].

Although the provision of the Finnish maternity health clinic services is dictated by law, the organisational model of these clinics is not. Thus, great structural diversity exists [13,16]. According to a recent survey, municipalities have organised maternity health clinic services mainly in three ways: clinics focusing solely on maternity care (16%); those combined with family planning services (33%); or those integrated with the child health clinics in which the same public health nurse cares for a family from the pregnancy until the child reaches school age (20%). Furthermore, various mixed models for maternity health clinics were implemented in 31% of the municipalities [13].

Critical discussion regarding the best model for maternity health clinics has been going on in Finland for long. Proposals for organisational development have concentrated on two main lines: promoting maternity health clinics that focus solely on reproductive health care or preferring maternity health clinics integrated into children’ health services. The reproductive-centred maternity health clinic model has been advocated e.g., by the fact that in these specialised clinics, nurses and physicians can focus particularly on women’s health issues and have adequate annual experience with pregnant clients. By providing all sexual and reproductive health services at the same clinic, women’s health could be comprehensively promoted. [17,18] On the other hand, the long-term continuity of care which is enabled by the integration of maternity and child health clinic services creates a propitious basis for the trustful relationship between professionals and the whole family, especially when psycho-social or other multidimensional problems arise. The integrated maternity and child health clinic has also been seen as a more father acknowledging and family-centred way to produce maternity care services than the separate maternity health clinic [19,20].

However, because of lack of evidence on how different organisational models for maternity health clinics influence the utilisation of the maternity care services as well as maternal and perinatal health outcomes, robust grounds for or against one single model for maternity health clinics could not have been presented. This study was carried out with the aim that it would for its part fill this knowledge gap.

The aim of this study was to compare maternity health clinics and the integrated maternity and child health clinics, in relation to selected outcomes obtained from the Finnish Medical Birth Register (Table 1). The research questions were:

Is there a relationship between the organisational model of the maternity health clinic and:

1. The utilisation of maternity care services (timing and number of the antenatal visits, antenatal screenings, hospitalisation during pregnancy)
2. Maternal outcomes (pregnancy and delivery-related outcomes of the woman)
3. Perinatal outcomes (health outcomes of the infant)

**Table 1:** Outcome measures with descriptive statistics used in the study.

Outcome*	Statistics

**Utilisation of maternity care services**	
First maternity care visit (gestational weeks)	Mean, SD
Visits in hospital maternity clinic	Mean, SD
Visits in maternity health clinic	Mean, SD
All maternity care visits during pregnancy	Proportion (n, %)
Late first maternity care visit (>15 gestational weeks)	Proportion (n, %)
Underutilisation of maternity care (1–5 visits)	Proportion (n, %)
Overutilisation of maternity care (>17 visits)	Proportion (n, %)
Serum screening for foetal abnormalities	Proportion (n, %)
Ultrasound screening for foetal abnormalities	Proportion (n, %)
Glucose tolerance test done	Proportion (n, %)
Hospital care during pregnancy	Proportion (n, %)
**Maternal outcomes**	
Gestational age at the time of delivery (gestational weeks)	Mean, SD
Pre-eclampsia^1^	Proportion (n, %)
Diabetes^2^	Proportion (n, %)
Duration of delivery (minutes)	Mean, SD
Method of delivery	
Vaginal	Proportion (n, %)
Breech birth	Proportion (n, %)
Vacuum or forceps	Proportion (n, %)
Section (includes elective and non-elective sections)	Proportion (n, %)
Induction	Proportion (n, %)
Pain relief in delivery	
Epidural	Proportion (n, %)
No medical pain relief (delivery with no medical pain relief method)	Proportion (n, %)
Physiological birth (vaginal birth with no medical pain relief and with no medical procedures^3^)	Proportion (n, %)
Episiotomy	Proportion (n, %)
Length of stay in hospital for mother (days)	Mean, SD
**Perinatal outcomes**	
Baby’s birth weight (g)	Mean, SD
Baby’s birth height (cm)	Mean, SD
Low Apgar score (5 min.)	Proportion (n, %)
Premature birth (birth before full 32 and full 37 weeks of gestation)	Proportion (n, %)
Small for gestational age (SGA, according to Finnish sex-specific standards)	Proportion (n, %)
Asphyxia	Proportion (n, %)
Intensive care or monitoring	Proportion (n, %)

^*^ Data from the Finnish Medical Birth Register 1 Jan 2009–31 Dec 2009. ^1^ Hypertension, proteinuria and oedema related to pregnancy and childbirth. ^2^ Abnormal glucose tolerance test during pregnancy. ^3^ Medical procedures recorded in the Finnish Medical Birth Register = induction, amniotomy, oxytocin, prostaglandin, amnioninfusion, episiotomy, manual removal of the placenta, evacuation of the uterus, suturing of the perineal trauma (degrees III–IV), mother transferred from another hospital, testing of the foetal blood pH, mother given blood transfusion during delivery.

This study is part of a broader study focused on developing the maternity and child health care services in Southwest Finland. The results of a previous phase of the research exploring the parents’ experiences and wishes regarding maternity and child health clinic services produced by different models have been reported elsewhere. [21,22].

## Methods

### Design and sample

A comparative, register-based cross-sectional design was used. The study was part of the multidisciplinary STEPS study that is being carried out in the catchment area of the Turku University Hospital by the Institute for Child and Youth Research at the University of Turku. This prospective STEPS study is based on a cohort of all Finnish or Swedish speaking women who had live deliveries in the Hospital District of Southwest Finland from January 2008 to April 2010 (N = 9811) and their children (N = 9936). Women who were unable to communicate in Finnish or Swedish were excluded (N = 661). The STEPS study protocol was approved by the Ethical Committee of the Turku University Hospital in June 2007 and by the Ministry of Social Affairs and Health in April 2008. The STEPS study protocol has been previously reported in greater detail by Lagström et al. [23].

**Figure 1:**
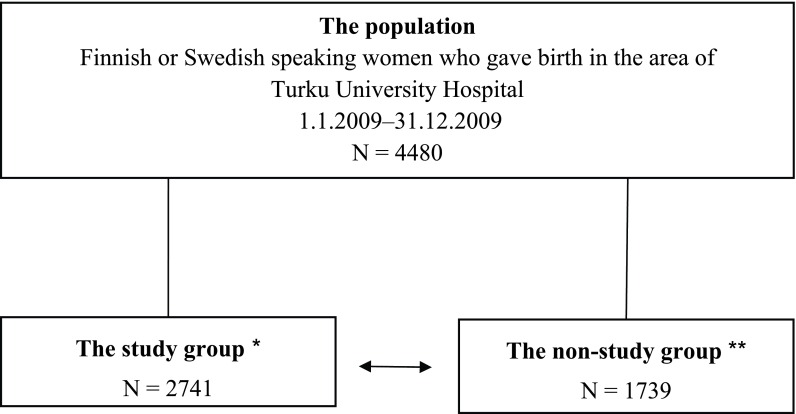
The formation of the study group. Information gathered from the Finnish Medical Birth Register. ^*^ Model of the woman’s maternity health clinic was known. ^**^ Model of the woman’s maternity health clinic was unknown.

The present data were collected as part of the STEPS study from the Medical Birth Register which is administered by the National Institute for Health and Welfare. The Medical Birth Register contains high-quality, complete information on the live births and stillbirths of more than 22 weeks of gestation or the baby weighing at least 500 grams in Finland since 1987. [24]. The data included Finnish or Swedish speaking women who gave birth in the area of Turku University Hospital between 1 January 2009 and 31 December 2009 (N = 4480). Additionally, it was required that the organisational model of the maternity health clinic that the women used was known. Due to the lack of this information the data of 1739 women had to be excluded. Finally the study group included a total of 2741 women (Figure 1). The background characteristics of the mothers in the study group were compared with the data of the excluded mothers to ensure representativeness of the study group.

### Measures

The information regarding the organisational models of the maternity health clinic services was gathered through a survey sent to the administrators of the health-care centres in the Turku University Hospital area in the spring of 2010. The administrators were asked whether the maternity health clinics were carried out separately or integrated with the child health clinics during the years 2008 and 2009. The necessary information was received from all the health centres covering the maternity health clinic units of 28 municipalities. Data from three small municipalities had to be excluded because of the municipalities’ structural changes (unification of municipalities and/or establishing of new health care consortiums) during the data collection which led to lack of exactly interpretable information regarding the organising of maternity health clinics. The municipalities with several maternity health clinic units organised under various models were excluded (N = 6), except one large municipality, which could be included because of more detailed information being available on each of the maternity health clinics and their clients, based on personal identification numbers. In addition, due to the later changes of women’s place of domicile, the information of 21 women is missing from comparative clinic’s model based analysis.

Information regarding the organisational model of a maternity health clinic was linked to the Medical Birth Register data based on the women’s place of domicile. For the comparative analysis, the data were classified into two groups according to the model of the women’s maternity health clinics: maternity health clinic or integrated maternity and child health clinic. The determinant was the maternity health clinic’s connection to a child health clinic’s services. The maternity health clinics that were linked to other primary health care services, such as family-planning clinics or school health care, were classified as maternity health clinics.

The outcome measures of the study were based on the Medical Birth Register data. The information regarding the women’s personal data, obstetric history, present pregnancy and delivery and its monitoring, as well as the baby’s health after delivery, were used. The outcome measures of the study are presented in Table 1.

### Data analysis

The data was analysed statistically using SPSS 20 and SAS Release 9.2. for Windows. The limit for statistical significance was set at p < 0.05. For continuous outcomes the comparative analysis between maternity health clinic models was conducted using a *t-*test (unadjusted) and ANOVA with a covariate (adjusted). For categorical outcomes the comparisons were conducted using Pearson’s chi-square / Fisher’s exact test (unadjusted) and logistic regression analysis with a covariate (adjusted). A statistical power analysis was performed for selected significant outcomes (= nulliparity).The effect of the organisational model of the maternity health clinic on outcome variables was adjusted by taking the significant background variables simultaneously as covariates to the analysis of variance model.

## Results

### Sociodemographic background of the participants

The essential sociodemographic and obstetric background variables of the study group and the non-study group (a cohort of parturients in Southwest Finland) are presented in Table 2. Comparisons showed that the study group was representative of the non-study group in relation to most of the examined background variables. However, there were more nulliparae in the study group than in the non-study group (p = 0.003). Moreover, women in the study group were more often intoxicant abusers (N = 54, 2.0% vs. N = 20, 1.2%, p = 0.036) and have given birth more often in a university hospital than women in the non-study group (N = 2529, 92.3% vs. N = 1202, 69.1%, p = <0.001).

**Table 2:** Background characteristics of the women. A comparison between the study group and non-study group and between maternity health clinic and integrated maternity and child health clinic.

	Study group^3^ N = 2741	Non- study group^4^ N = 1739	p^*^	Maternity health clinic N = 2178	Integrated maternity and child health clinic N = 542	p^*^

Age	n (%)	n (%)		n (%)	n (%)	
	
mean, years (SD)	30.4 (4.988)	30.2 (5.182)	0.279	30.1 (5.196)	30.7 (5.073)	0.010
min-max (years)	16.5–49.6	16.7–47.6		16.5–49.6	18.7–45.1	
< 18	12 (0.4)	6 (0.35)	0.732	12 (100)	0 (0)	0.077
> 35	462 (16.9)	306 (17.6)		356 (77.4)	104 (22.6)	
**Civil status**			0.386			0.790
Married	1502 (54.8)	971 (55.8)		1192 (54.7)	304 (56.1)	
Unmarried	1208 (44.1)	755 (43.4)		961 (44.1)	233 (43.0)	
Other	31 (1.1)	13 (0.8)		25 (1.2)	5 (0.9)	
**Nulliparity**			0.003^**^			<0.001^***^
(= no previous births)	1278 (46.6)	732 (42.1)		1053 (48.3)	212 (39.1)	
**BMI**						
>30	339 (12.5)	234 (13.6)	0.270	267 (12.3)	72 (13.4)	0.220
<19	156 (5.8)	83 (4.8)		133 (6.1)	23 (4.3)	
**Previous abortion**	394 (14.4)	258 (14.9)	0.665	306 (14.1)	86 (15.9)	0.285
**Intoxicant abuse**	53 (2.0)	20 (1.2)	0.036	44 (2.0)	9 (1.7)	0.588
**Smoking during pregnancy**	484 (17.8)	291 (16.7)	0.173	386 (17.8)	98 (18.1)	0.852
**Fertilisation treatment**^1^	53 (2.0)	34 (2.0)	0.838	33 (1.5)	20 (3.7)	0.001
**Delivery in university hospital**^2^	2529 (92.3)	1202 (69.1)	<0.001	2015 (92.6)	494 (91.1)	0.285
**Method of delivery**			0.951			0.842
Vaginal	2104 (76.8)	1345 (77.4)		1675 (76.9)	418 (77.1)	
Breech birth	23 (0.8)	16 (0.9)		17 (0.8)	6 (1.1)	
Vacuum or forceps extraction	245 (8.9)	152 (8.7)		197 (9.0)	45 (8.3)	
Section	369 (13.5)	226 (13.0)		289 (13.3)	73 (13.5)	
**Twins/triplets**	42 (1.5)	34 (2.0)	0.332	29 (1.3)	13 (2.4)	0.059

Information gathered from the Finnish Medical Birth Register. ^1^ In vitro fertilisation, artificial insemination or ovulation induction. ^2^ <8000 births/year. ^3^ Women who gave birth in the area of Turku University Hospital between 1 Jan 2009–31 Dec 2009 and their maternity health clinic’s model was known. ^4^ Women who gave birth in the area of Turku University Hospital between 1 Jan 2009–31 Dec 2009 and their maternity health clinic’s model was unknown. ^*^ Used statistical test: Pearson’s Chi Square. ^**^ A statistical power analysis: alpha = 0.05, power = 0.845. ^***^ A statistical power analysis: alpha = 0.05, power = 0.987.

Furthermore, the women’s background characteristics in relation to the model of the maternity health clinic were explored. There were no significant differences between the clinic models regarding most of the background characteristics, except the number of nulliparous women, which was greater in the maternity health clinics than the integrated maternity and child health clinics (N = 1053, 48.3% vs. N = 212, 39.1%, p = <0.001). In addition, in integrated clinics the mean age of women was higher (30.7 years vs. 30.1 years, p = 0.010) and they had undergone more fertilisation treatments than the women in the separate maternity health clinics (N = 20, 3.7% vs. N = 33, 1.5%, p = 0.001).

### The relation between the model of maternity health clinic and utilisation of maternity care services

The majority of the studied women had used the services of the maternity health clinics (N = 2178, 80.1%), with a fifth (N = 542, 19.9%) having used the integrated maternity and child health clinics. The relationship between the model of the maternity health clinic and the utilisation of maternity care services is described in Table 3. The comparison of the models indicated that women who had used the services of a maternity health clinic had their first maternity care visit earlier than women who had used the services of an integrated maternity and child health clinic. They also more frequently visited a hospital maternity outpatient clinic than the women who had used an integrated maternity and child health clinic. Furthermore, the glucose tolerance test was conducted more often on women who had used a separate maternity health clinic. Accordingly, hospital care during pregnancy was more common with women who had used the services of a separate maternity health clinic. The differences in the hospitalisation between the groups occurred generally and with one specified reason for the hospital care.

**Table 3:** Utilisation of maternity care in relation with the organisational model of the maternity health clinic.

Outcome	Maternity health clinic N = 2178	Integrated maternity and child health clinic N = 542	Unadjusted	Adjusted^*^

	Mean (SD)	Mean (SD)	p	p
First maternity care visit (gestational weeks)	8.8 (2.649)	9.5 (2.737)	<0.001	0.003
Visits in hospital maternity clinic	2.87 (2.508)	2.61 (2.170)	0.027	0.769
Visits in maternity health clinic	11.57 (3.575)	11.72 (3.062)	0.423	
All maternity care visits during pregnancy	14.42 (4.205)	14.35 (3.504)	0.681	
	**n (%)**	**n (%)**	**p**	**p**

Late first maternity care visit (>15 gestational weeks)	41 (1.9)	14 (2.6)	0.301	
Underutilisation of maternity care (1–5 visits)	25 (1.1)	5 (0.9)	0.656	
Overutilisation of maternity care (>17 visits)	549 (25.2)	130 (24.0)	0.571	
Serum screening for foetal abnormalities	100 (4.6)	16 (3.0)	0.091	
Ultrasound screening for foetal abnormalities	2149 (98.7)	531 (98.0)	0.227	
Glucose tolerance test done	1352 (62.1)	304 (56.1)	0.011	0.386
Hospital care during pregnancy	145 (6.7)	21 (3.9)	0.015	0.124
Bleeding	13 (0.6)	1 (0.2)	0.327	
High blood pressure	11 (0.5)	4 (0.7)	0.517	
Prematurity	16 (0.7)	2 (0.4)	0.553	
Other reason	115 (5.3)	15 (2.8)	0.014	0.067

Used statistical tests: Continuous outcomes: T-test (unadjusted) and Analysis of variance with covariates (adjusted). Categorical outcomes: Pearson’s Chi Square/Fisher’s exact test (unadjusted) and Logistic regression analysis with covariates (adjusted). ^*^ Adjusted with nulliparity, age and fertilisation treatment.

### The relation between the model of maternity health clinic and maternal and perinatal outcomes

There were no statistically significant differences between the maternity health clinic and integrated maternity and child health clinic models regarding the majority of the explored delivery and infant related outcomes (Table 4). However, the lengths of both the first stage of delivery (802.2 min vs. 727.9 min, p = 0.011) and the second stage (35.3 min vs. 31.3 min, p = 0.054) were longer for the women who had used a maternity health clinic than the women in the integrated maternity and child health clinic group. Women who had used a maternity health clinic were more likely to have epidural analgesia as pain relief during delivery (57.6% vs. 48.9%, p = < 0.001) and they had more episiotomies (11.2% vs. 8.1%, p = 0.037) than the women who had used an integrated maternity and child health clinic. Delivery without any pain relief was more common with women in the integrated clinic group (20.9% vs. 17.2%, p = 0.049). Furthermore, the baby’s birth weight was greater in those born to mothers in the integrated clinic group than the maternity clinic group (3507.5 g vs. 3497.4 g, p = 0.038).

**Table 4:** Maternal and perinatal outcomes in relation with the organisational model of the maternity health clinic.

Outcome	Maternity health clinic N = 2178	Integrated maternity and child health clinic N = 542	Unadjusted	Adjusted^*^

**Maternal**	**Mean (SD)**	**Mean (SD)**	**p**	**p**
Gestational age at the time of delivery (weeks)	39.7 (1.976)	39.7 (1.870)	0.796	
Duration of delivery (min)				
First stage	802.2 (551.085)	727.9 (531.159)	0.011	0.563
Second stage	35.3 (40.833)	31.3 (36.675)	0.054	0.186
Length of stay in hospital for mother (days)	4.0 (2.082)	3.9 (2.086)	0.314	
	**n (%)**	**n (%)**	**p**	**p**
	
Pre-eclampsia ^1^	129 (5.9)	36 (6.6)	0.530	
Diabetes ^2^	316 (14.5)	76 (14.0)	0.773	
Method of delivery				
Vaginal	1675 (76.9)	418 (77.1)	0.842	
Breech birth	17 (0.8)	6 (1.1)		
Vacuum or forceps	197 (9.0)	45 (8.3)		
Section (elective and non-elective sections)	289 (13.3)	73 (13.5)		
Induction	400 (18.4)	94 (17.3)	0.581	
Pain relief in delivery				
Epidural	1254 (57.6)	265 (48.9)	>0.001	0.148
No medical pain relief	373 (17.2)	113 (20.9)	0.049	0.518
Physiological birth^3^	612 (28.5)	170 (31.4)	0.191	
Episiotomy	244 (11.2)	44 (8.1)	0.037	0.099
**Perinatal**	**Mean (SD)**	**Mean (SD)**	**p**	**p**

Baby’s birth weight (g)	3497.4 (571.879)	3507.5 (553.323)	0.038	0.167
Baby’s birth height (cm)	50.7 (2.481)	50.9 (2.321)	0.107	
	**n (%)**	**n (%)**	**p**	

Low Apgar score (Apgar score 0–6)	45 (2.1)	14 (2.6)	0.461	
Premature birth				
birth before full 37 weeks of gestation	90 (4.2)	20 (3.8)	0.630	
birth before full 32 weeks of gestation	17 (0.8)	3 (0.6)	0.781	
Small for gestational age (SGA)	46 (2.1)	7 (1.3)	0.216	
Asphyxia	171 (7.9)	34 (6.3)	0.213	
Baby’s intensive care or monitoring	226 (10.4)	59 (10.9)	0.729	

^1^ Hypertension, proteinuria and oedema related to pregnancy and childbirth. ^2^ Abnormal glucose tolerance test during pregnancy. ^3^ Vaginal birth with no medical pain relief and with no medical procedures. Used statistical tests: Continuous outcomes: T-test (unadjusted) and Analysis of variance with covariates (adjusted). Categorical outcomes: Pearson’s Chi Square/Fisher’s exact test (unadjusted) and Logistic regression analysis with covariates (adjusted). ^*^ Adjusted with nulliparity, age and fertilisation treatment.

### Results of the adjusted analysis

Because the women were older and more often nulliparae and had undergone fertilisation treatment in the maternity health clinic group, the age, nulliparity and fertilisation treatment were chosen to be the confounding background variables when comparing the outcomes by the model of maternity health clinic. In this adjustment model, nulliparity [F(1, 2709) = 6.43, p = 0.011], age [F(1, 2709) = 5.22, p = 0.022] and fertilisation treatment [F(1, 2709) = 14.73, p = 0.001], explained the effect that the clinic model had on the frequency of visits to a hospital out-patient maternity clinic. In addition, nulliparity [F(1, 2711) = 60.08, p = <0.001], age [F(1, 2711) = 72.94, p = <0.001] and fertilisation treatment [F(1, 2711) = 4.65, p = 0.031] explained the effect that the clinic model had on the frequency of the glucose tolerance tests. The timing of the first antenatal visit of women who had used the services of a separate maternity health clinic, earlier in pregnancy, was explained by both the model of the clinic [F(1, 2705) = 8.84, p = 0.003], age [F(1, 2705) = 11.21, p = 0.001) and nulliparity [F(1, 2705) = 4.04, p = 0.044]. The impact of the clinic model on general hospitalisation during pregnancy was explained by fertilisation treatment [F(1, 2710) = 8.54, p = 0.004] and as well on hospitalisation because of “other reasons” [F(1, 2710) = 6.63, p = 0.010].

Moreover, the nulliparity explained the clinic model’s effect on the length of the first stage [F(1, 2284) = 264.51, p = < 0.001] and second stage [F(1, 2284) = 391.55, p = < 0.001] of delivery together with age [F(1, 2284) = 13.48, p = 0.001]. The clinic model’s effect on the baby’s birth weight was explained by nulliparity [F(1, 2712) = 42.98, p = < 0.001] and fertilisation treatment [F(1, 2712) = 8.00, p = 0.005]. Differences between the groups regarding birth without pain relief were also explained by nulliparity [F(1, 2720) = 18.07, p = < 0.001] and age [F(1, 2720) = 24.65, p = < 0.001]. Nulliparity [F(1, 2720) = 171.16, p = <0.001] and age [F(1, 2720) = 4.02, p = 0.045] also explained the clinic model’s effect on the frequency of epidural analgesia during delivery. The clinic model’s effect on the frequency of episiotomy was explained by nulliparity [F(1, 2720) = 89.76, p = < 0.001].

Finally, the organisational model of the maternity health clinic was not found to be related to service utilisation outcomes, with the exception of the first maternity care visit, or to any maternal or perinatal outcomes.

## Discussion

The findings of this study indicate that the organisational model of the maternity health clinic does not have a notable impact on the utilisation of maternity care services. Moreover, there were no differences between the maternity health clinic and integrated maternity and child health clinic models concerning the explored birth- and infant-related health outcomes. From the perspective of the health service system, this means that desirable results in terms of perinatal health could be achieved with both integrated and separate maternity health clinic models. This is in line with evidence from other European countries [6,15] and from Australia [25], which indicates that effective and safe maternity care can be delivered within diverse organisational settings. Hence, a coherent understanding about the best possible structure for maternity health clinic services still remains elusive.

If equally good health outcomes can be produced through various maternity health clinic models—as our results suggest—the clients’ experiences and wishes should be emphasised when assessing the quality of different service models. Our previous findings indicate that the integrated maternity and child health clinic model that enables long-term relational continuity of care between the same public health nurse and family improve parents’ service experiences. For example, the integrated clinic model seems to provide more home visits and support for the families than the separate maternity health clinic. [21,22]. Furthermore, there is some evidence that Finnish parents would rather use the integrated maternity and child health clinic than the separate clinics [20]. It is assumed that this could be a consequence of trust and familiarity fostered in a long-term relationship between the nurse and the family that is made possible in the integrated maternity and child health clinics. According to the review of Sandall et al. [12], the continuity of midwifery care increases women’s satisfaction with maternity care. Similarly, the review of Britton [26], which focused on the satisfaction of care during the perinatal period, suggested that the essential determinants of families’ health service experiences seem to be the relationship between the caregiver and family, and the families’ perceptions of the support and information provided. Thus, it can be recommended that these aspects evaluated from the perspective of parents should form the basis for the organisational strategies of maternity and child health clinic services.

The renewed National Development Programme for Social Welfare and Health Care (Kaste) calls for social welfare and health-care services to be organised in a client-oriented and economically sustainable way [27]. In the field of maternity and child health care, this means that the cost-effectiveness of different maternity and child health clinic models should be rigorously measured. Due to a lack of evidence, comparative research into the economic influences of separate maternity health clinic and integrated maternity and child health clinic, as well other models, is thus crucial. In addition to the economic evaluation, the effectiveness of health promotion provided by maternity and child health clinics should be set in the scope of future research. Although several researchers have explored health promotion within the Finnish maternity and child health clinic system such as the dietary and physical activity counselling [28,29,30,31], support for breastfeeding [32], and support for mothers’ post-natal mental health [33], they have not considered the impact of different clinic models on implementing these health promotions. Thus, the long-term evaluation of the effects of health promotion provided by diverse maternity and child health clinic settings would be beneficial [34].

Our results provide novel views to the discussion regarding the organisation of primary maternity and child health care services. Experts in Finland have not yet reached an agreement on whether maternity health clinics should be organised as separate clinics focusing on women’s reproductive health issues [17] or integrated with children’s and families’ health and welfare services [19]. It has been assumed that an increased amount of tertiary-level maternity care might be a consequence of the maternity health clinics’ fragmented organisational structure and the varying professional background of the maternity health clinics’ personnel [17,35]. In addition, concern has been expressed by experts about the sufficient obstetric competence of public health nurses whose work pattern in a maternity health clinic includes primary health care tasks beside those relating to maternity care [17,18,35,36]. This debate has also been represented in public discussions in the media. However, in contrast to these concerns, our results indicate that the model of an integrated maternity and child health clinic, which provides both maternity and child health care, was not associated with more frequent visits to a hospital maternity clinic or an increased likelihood of hospitalisation during pregnancy than the model of a separate maternity health clinic. A study exploring maternal and perinatal outcomes in relation to the professional education and competence of maternity health clinic nurses and physicians would shed further light on this discussion.

The strength of our study is that it produced the first comparative register-based report of the outcomes of separate maternity health clinic and integrated maternity and child health clinic models in Finland. The comparison was based on the existing structure of the Finnish maternity and child health clinic system which provides, because of its natural variation, a favourable field for comparative health-service research without requiring complex experimental settings to be built. In addition, the outcome measures of the study were based on the routinely collected register data of the Medical Birth Register. Consequently, the reliability and cost-effectiveness of the study can be evaluated as good. Furthermore, national registers with personal identification numbers have previously proved to be reliable and cost effective sources for comparative health-care service research [37].

Our study focused on the area of the Turku University Hospital. Despite the Finnish population being homogenous to a large extent, the national generalisability of our results should be considered. Because national evidence on the impact of the maternity health clinic models on maternal and perinatal outcomes is still lacking, an inclusive nation-wide register-based comparison of different maternity health clinic models is necessary to draw firm conclusions. However, the problem remains that no national registers, including the Medical Birth Register, currently include information regarding the model of maternity health clinic that the women use. Thus, implementing a national comparison is complex and would require extensive gathering of data from health centres. In the future, the collection of data regarding the model of maternity health clinic services could be contingently linked to the maintained primary health-care statistics Avohilmo that has, since 2011, collected national information on the availability, content and users of primary health-care services [38]. This linkage would also ease the comparison of the costs of different maternity health clinic models which would provide very important information for policy makers and public health service organisers. Currently, there is no comparative evidence regarding the cost-effectiveness of separate maternity health clinics and integrated maternity and child health clinics in Finland. Moreover, the lack of detailed sociodemographic background variables of women available from the Medical Birth Register may be considered a limitation of the study.

## Conclusions

Our regional study indicates that the model of maternity health clinic does not have a clinically significant effect on the utilisation of maternity care services. It also seems that equally good maternal and perinatal outcomes can be achieved within separate maternity health clinic and integrated maternity and child health clinic settings. Primary maternity care could thus be provided with similar outcomes either in a separate maternity health clinic or integrated to child health services. A larger, nation-wide data set is needed to confirm the findings of this study. Alongside the utilisation and health indicators, families’ wishes and experiences should be consi dered when making decisions regarding the organisation of maternity and child health clinic services.
